# Two Singapore public healthcare AI applications for national screening programs and other examples

**DOI:** 10.1002/hcs2.10

**Published:** 2022-08-19

**Authors:** Andy Wee An Ta, Han Leong Goh, Christine Ang, Lian Yeow Koh, Ken Poon, Steven M. Miller

**Affiliations:** ^1^ Department of Data Analytics and AI Integrated Health Information Systems (IHiS) Private Limited Singapore Singapore; ^2^ School of Computing and Information Systems Singapore Management University Singapore Singapore

**Keywords:** AI applications, AI for national screening programs, influence of clinical context on AI design and usage

## Abstract

This article explains how two AI systems have been incorporated into the everyday operations of two Singapore public healthcare nation‐wide screening programs. The first example is embedded within the setting of a national level population health screening program for diabetes related eye diseases, targeting the rapidly increasing number of adults in the country with diabetes. In the second example, the AI assisted screening is done shortly after a person is admitted to one of the public hospitals to identify which inpatients—especially which elderly patients with complex conditions—have a high risk of being readmitted as an inpatient multiple times in the months following discharge. Ways in which healthcare needs and the clinical operations context influenced the approach to designing or deploying the AI systems are highlighted, illustrating the multiplicity of factors that shape the requirements for successful large‐scale deployments of AI systems that are deeply embedded within clinical workflows. In the first example, the choice was made to use the system in a semi‐automated (vs. fully automated) mode as this was assessed to be more cost‐effective, though still offering substantial productivity improvement. In the second example, machine learning algorithm design and model execution trade‐offs were made that prioritized key aspects of patient engagement and inclusion over higher levels of predictive accuracy. The article concludes with several lessons learned related to deploying AI systems within healthcare settings, and also lists several other AI efforts already in deployment and in the pipeline for Singapore's public healthcare system.

## Graphical abstract

This article explains how two AI systems have been incorporated into the everyday operations of two Singapore public healthcare nation‐wide screening programs. Ways in which healthcare needs and the clinical operations context influenced the approach to designing or deploying the AI systems are highlighted, illustrating the multiplicity of factors that shape the requirements for successful large‐scale deployments of AI systems that are deeply embedded within clinical workflows.

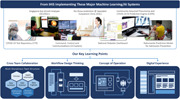

**Abbreviations**
BRAIN: the Business Research Analytics Insight Network platform, a centralized business intelligence, analytics, and AI processing platform built by IHiS to serve a wide range of data analytics needs across Singapore's public healthcare system
CCMS: Care and Case Management System
GP: general practitioner
HDG: healthcare data grid
H2H: “hospital to home” national program to provide a common nation‐wide community care model targeted at the discharged patients—almost all elderly—with the most complex health and related social support needs
IHiS: Integrated Health Systems, the Singapore national healthtech agency
NEHR: National Electronic Health Record system
SELENA+: Singapore Eye LEsioN Analyzer deep learning AI system used within the SiDRP workflow
SiDRP: Singapore Integrated Diabetic Retinopathy Program

## 1 THE SINGAPORE HEALTHCARE CONTEXT AND PRIORITIES FOR PUBLIC ENGAGEMENT

Singapore, a nation with a relatively small population of about 5.5 million people [1], is globally reputed for its excellent public healthcare services. Yet, the country is already experiencing increasingly strong pressures on the public healthcare system due to demographic trends. These will only intensify over time.

Given this context, the Singapore government is aggressively reshaping its approach to public healthcare by putting even more emphasis on efforts to motivate healthy living that would prevent or reduce disease occurrence, and by substantially expanding national screening efforts to uncover and address chronic disease and related complications earlier [2]. At the same time, there are numerous ongoing initiatives to improve the productivity and quality of healthcare service delivery in the public hospitals and network of community care clinics in order to increase capacity and quality of care in these facilities. To this end, the public healthcare service delivery institutions and support agencies, in partnership with the national healthtech agency Integrated Health Information Systems (IHiS), have been developing and deploying various AI models to support a wide range of patient‐centric care initiatives.

This article highlights two AI applications that are widely used across Singapore's public healthcare system for different types of national screening programs. The first example is embedded within the setting of a national level population health screening program for diabetes related eye diseases, targeting the rapidly increasing number of adults in the country with diabetes. In the second example, the AI assisted screening is done shortly after a person is admitted to one of the public hospitals to identify which inpatients—especially which elderly patients with complex conditions—have a high risk of being readmitted as an inpatient multiple times in the months following discharge. Additional AI applications beyond these two are listed in Table 1 and discussed in the final section on “Moving Forward at the National Level and Lessons Learned.”

**Table 1:** Sample of AI enabled systems already in use (A) or in the pipeline (B) in Singapore's public healthcare system for supporting early detection/prevention, treatment targeting, resource optimization, and administration

Purpose of AI enabled system	Stage of usage	Brief description of AI application
(1) Early‐stage detection and prevention	(A) Already deployed at scale nationally	SELENA+ system for analyzing eye retina images to detect three types of eye disease in support of the national diagnostic retinopathy screening program (described in this article).
	(B) In the pipeline∗	Identification of diabetic patients at risk of kidney failure through the National Diabetes Dashboard and the underlying National Diabetes Database. Prediction of the risk of ischemic heart disease using data from the National Diabetic Database and other data sources. Risk assessment tools for chronic disease management for primary care providers (GPs, family doctors, community clinics) to use for their patient assessments. Prediction of the risk of severe bone loss from cone beam computed tomography.
(2) Targeting for clinical treatments as well as targeting for related special health support programs	(A) Already deployed at scale nationally	Prediction of hospital inpatients with a high risk of multiple readmissions in support of the hospital to home national program (described in this article).
	(B) In the pipeline∗	Prediction of mortality using chest radiographs for patients with community‐acquired pneumonia. Prediction of adverse drug reactions to assist decisions on medication as part of mitigating inpatient readmission risk. Recommendation to assist determination of dosage titration for using the antibiotic vancomycin [40].
(3) Resource optimization within hospitals and polyclinics, and across the entire public healthcare supply chain and service delivery network	(A) Already deployed at scale at one large public hospital, and in the process of being implemented at other hospitals	Comprehensive Command and Control Center (C3) with supporting AI models to predict
		Short term and near‐term emergency department arrivals. Specialist outpatient clinic patient loads on a month‐to‐month basis. Number of people entering and leaving hospital via all entry points, and number of people in key hospital areas susceptible to crowd build ups via real‐time video analytics.
		C3 center uses various AI enhanced simulation models to support action planning and option assessment for dealing with current or impending congestion, load imbalances, and resource constraints across key hospital resources.
	(B) In the pipeline∗	Prediction of patient length of stay and discharge before noon for hospital bed management. Simulation of patient care end‐to‐end flows including flows through the hospital (through emergency department, admission to wards, and ward discharge, incorporating real‐time feeds of queue lengths, and other relevant historical and real time hospital operations data) and also flows into community care programs. Social media monitoring and tracking of new trends and hot topics across the globe to detect early signs of unusual disease outbreaks in order to detect possibilities of impending patient load surges at Singapore public hospitals.
(4) Administrative support	(A) Already deployed at scale nationally	Use of robotic process automation and wireless sensors to facilitate covid patient discharge from special facilities set up as covid community care facilities.
	(B) In the pipeline∗	Medical supplies inventory monitoring and tracking using deep learning imaging. Estimation of hospital inpatient bill size to facilitate and speed up patient discharge process.

∗ In the pipeline means either (i) in the process of large‐scale trials and/or early deployment, or (ii) under development and/or early‐stage pilot testing.

## 2 NATIONAL LEVEL SCREENING OF EYE RETINA IMAGES FOR DIABETES RELATED EYE DISEASES

### 2.1 Objectives

In Singapore, diabetes is a serious health concern, with over 400,000 Singaporeans living with the disease. One in three Singaporeans has a lifetime risk of getting diabetes and the number of those with diabetes is projected to reach one million by 2050, if current trends continue [3]. More current figures estimate the size of Singapore's diabetic population to be over 700,000 people, with over half of them not even knowing they currently have the disease [4].

This is an alarming situation as the progression of diabetes can lead to a wide range of serious health problems and place heavy loads on public hospitals. Through the “War on Diabetes” effort, national engagement has proceeded along three fronts: (i) prevention of diabetes‐related disease occurrence through promotion of healthier living, (ii) earlier detection and intervention through screening to catch problems before they result in more serious health burdens, and (iii) better disease management [5, 6]. There has also been supporting national R&D expenditures to improve capabilities for these three types of efforts.

Of particular concern is diabetic retinopathy, whereby blood vessels in the retina (inner nerve lining of the eyeball) are damaged by diabetic related complications, resulting in bleeding and fluid accumulation, leading to decreased vision. This condition is one of the most severe complications of diabetes.

Between 20% and 40% of all patients with diabetes end up with diabetic retinopathy, with 5%−10% of all diabetics ending up with severe vision threatening eye disease resulting in near or total blindness if their condition is left untreated [7, 8, 9]. These estimates of prevalence increase as a population ages [10]. As such, regular screening for diabetic retinopathy amongst all patients with diabetes is now recommended in international guidelines for care [11].

In this context, the objective of this AI application has been on making national level screening for diabetic related eye diseases and other eye diseases related to ageing more accessible to all adults, especially those aged 40 and older. As noted by the joint team of clinicians and researchers that developed this solution, their challenge and therefore objective was to create and implement a national level diabetic retinopathy screening program that would address both workforce constraint obstacles (due to the labor intensive nature of “reading” and interpreting the eye retina images) and the overall financial issues (cost per patient screened for a national level program)—which have been barriers encountered by other countries that have attempted this type of national screening effort [12].

### 2.2 AI application and total system

The origin of this overall effort consists of two tightly intertwined long term project efforts extending over the course of a 20‐year period. The first is the Singapore Integrated Diabetic Retinopathy Program (SiDRP), which centralized and standardized the review of eye retina images by trained human technicians, and the second is Singapore Eye LEsioN Analyzer (SELENA+), the deep learning system for analyzing eye retina images to detect the presence of diabetic retinopathy and several other age‐related eye diseases that was later incorporated into the SiDRP workflow.

#### 2.2.1 The SiDRP

**Figure 1:**
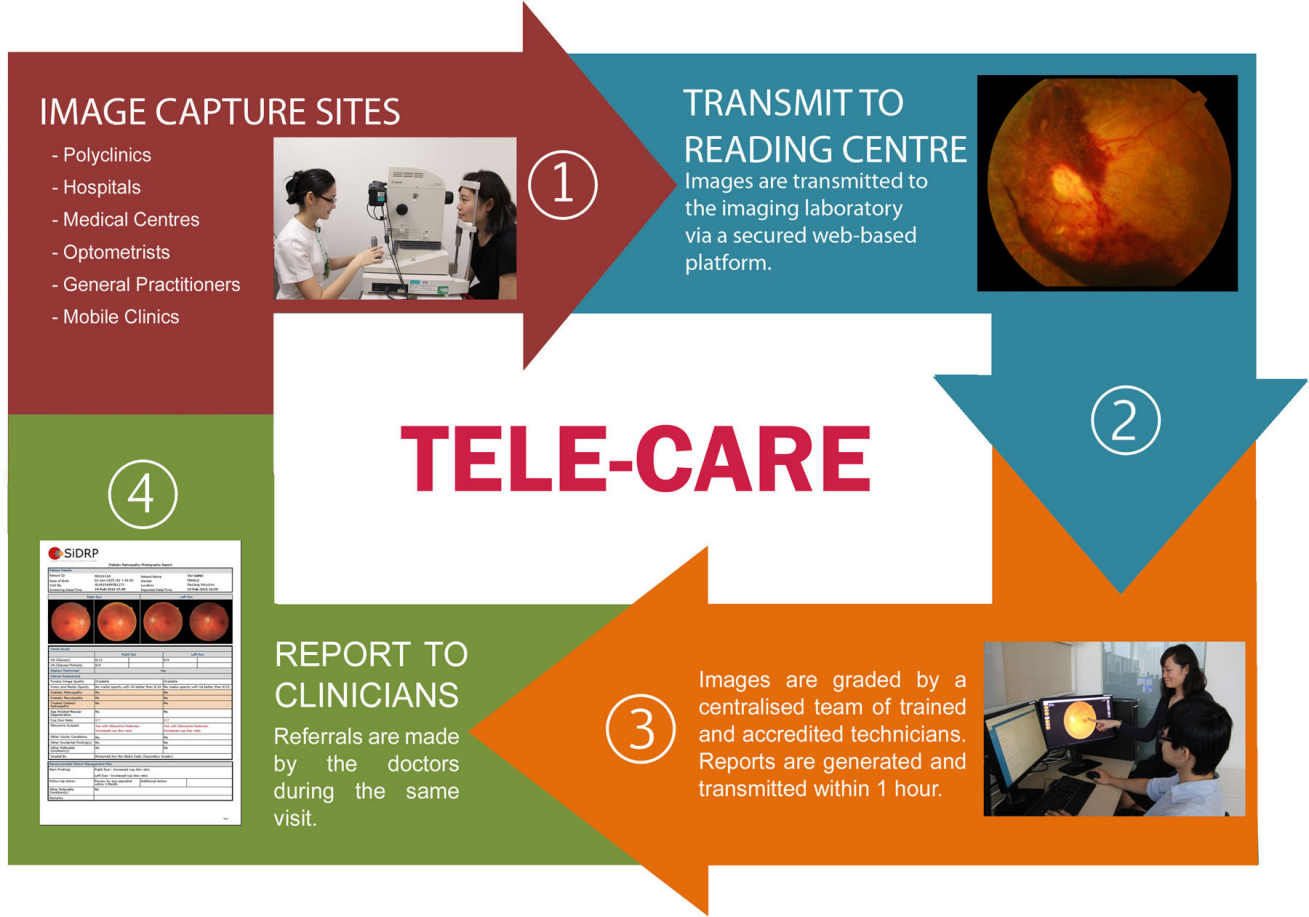
Summary of the Singapore Integrated Diabetic Retinopathy Program (SiDRP). *Source*: Singapore National Eye Center, SingHealth.

Building on a backbone of a national tele‐ophthalmology IT infrastructure for capturing eye images from a wide range of neighborhood screening centers, SiDRP was established in 2010 to scale up national screening specifically for diabetic retinopathy. This is part of the national engagement effort to encourage the public to go for this type of eye screening [13] (see Figure 1). At a neighborhood eye screening location where this service is set up, retinal images are captured from undilated pupils by a nurse practitioner using a specialized camera. Using the national tele‐ophthalmology platform, the digital retinal images are sent to the centralized reading center.

Prior to the incorporation of AI‐based image screening, all images were reviewed by a Level 1 trained primary reader and those showing signs of diabetic retinopathy were forwarded to a Level II more‐skilled secondary reader for confirmation. Audit checks of the human technician assessments were conducted regularly, and results showed 90% sensitivity (ability to correctly identify patients with a disease) and specificity (ability to correctly identify people without the disease) when measured against the “gold standard” of evaluation by a medical doctor ophthalmologist [14]. This entire screening effort—from capturing the eye image using the specialized camera at neighborhood health centers to centrally analyzing the images for signs of diabetic retinopathy—could be conducted by trained technicians and achieve what was considered to be a very high quality result. And this approach was scalable, starting with about 2200 annual screenings in 2010 and increasing to over 100,000 annual screenings by 2019 [15].

#### 2.2.2 SELENA+

Since the early 2000s, there had been an ongoing three‐way research collaboration involving the Singapore National Eye Center (clinical practitioners), the Singapore Eye Research Institute (medical researchers, some who were also clinical practitioners), and the School of Computing at the National University of Singapore (academic computer science and AI researchers) to develop software algorithms to automatically analyze eye images and predict the occurrence of several specific types of eye disease, including diabetic retinopathy. This longstanding collaboration substantially benefitted from the SiDRP effort started in 2010, as this provided image and evaluation data of much higher quality than previously available. It also benefited from the well‐known developments stemming from the ImageNet competitions in 2012 and 2013 when it became evident to the global AI research community and to industry AI leaders at that time that it was now possible to apply multilayer neural networks (deep learning models) to image analysis and achieve results that far surpassed capabilities of prior methods and accuracy levels of previous benchmarks [16].

The continuation of this three‐way research collaboration led to the development of the SELENA+ to analyze retinal images for signs of three major diabetic related eye diseases: diabetic retinopathy, glaucoma, and age‐related macular degeneration (AMD) [17]. SELENA+ is a deep learning convolutional neural network that implicitly recognizes characteristics of referable diabetic retinopathy, possible glaucoma, and AMD from appearances in retinal images [18]. The deep learning system is an ensemble model consisting of three deep neural networks (VGGNet, ResNet, and DenseNet) [12]. The system was initially trained on retinal images (with and without each of the three conditions) from the SiDRP collected between 2010 and 2013, and then validated and refined over the next several years with additional training and hold‐out data from SiDRP and from other populations.

Large scale field trials using SELENA+ in the context of SiDRP started in November 2018. Over a 1‐year period, more than 38,000 patient retinal photographs from six public community‐based polyclinics were processed by SELENA+ and reviewed at the centralized Ocular Reading Center. In parallel, these results were compared to the image grading performed by human technician readers. The accuracy of SELENA+ in detecting vision‐threatening diabetic retinopathy, glaucoma suspects, and late stage AMD was assessed as being comparable to the current standard of care [19]. This led to a second 1 year phase of field evaluation starting in December 2020 where the field trial was extended to an additional six public polyclincs to determine how to most appropriately incorporate SELENA+ into the existing SiDRP workflow and also to evaluate its performance compared to human graders for detecting mild to moderate diabetic retinopathy cases. Following this was a third stage, full scale deployment of SELENA+ to analyze eye images from all 23 of the public community‐based polyclinics and other community locations, starting in January 2022 [20].

Shortly before the start of the first phase field trial in November 2018, the core team involved in the development of SELENA+ formed a commercial entity called EyRIS [21]. This entity secured start‐up funding to handle the needs for the additional clinical device support the SELENA+ team would require to enhance and productize the AI engine for the upcoming large scale field trials, follow‐on full scale national deployment, and anticipated international expansion [22].

The SiDRP/SELENA+ field trials and full‐scale national deployment required a huge amount of integration across multiple subsystems and associated vendors. This included the cameras used to create the eye images at all the neighborhood screening clinic locations, the national tele‐ophthalmology network, the SELENA+ AI engine, the centralized Ocular Reading Center systems, linkages to the national hospital systems for referrals, and end‐to‐end data management. To handle this, in mid‐2018 IHiS (Singapore's National healthtech Agency) was brought into the effort to partner on the implementation with all public healthcare entities and private sector participants across the SiDRP/SELENA+ ecosystem.

IHiS handled procurement, vendor management, overall system integration and testing, end‐to‐end data management, the machine learning operations pipeline, and ongoing support for the total integrated system. It had to make sure the infrastructure was right sized for low latency response, that all data pipelines worked properly, that error handling was done in a systematic and user friendly way, and that the IT, data processing and data flow aspects of the end‐to‐end workflow were efficiently executed and smoothly integrated to achieve the service level requirement of delivering the screening result report back to the patient and care providers in under 1 hour.

In September 2020, EyRIS was awarded a 5‐year contract from IHiS, structured as an option to exercise on a yearly basis, to handle the ongoing support and enhancement of SELENA+ as the AI system was a key part of the national SiDRP [23]. The award announcement noted this would be a world‐wide first of using an approved AI “medical device” as a core part of a national health screening program.

**Figure 2:**
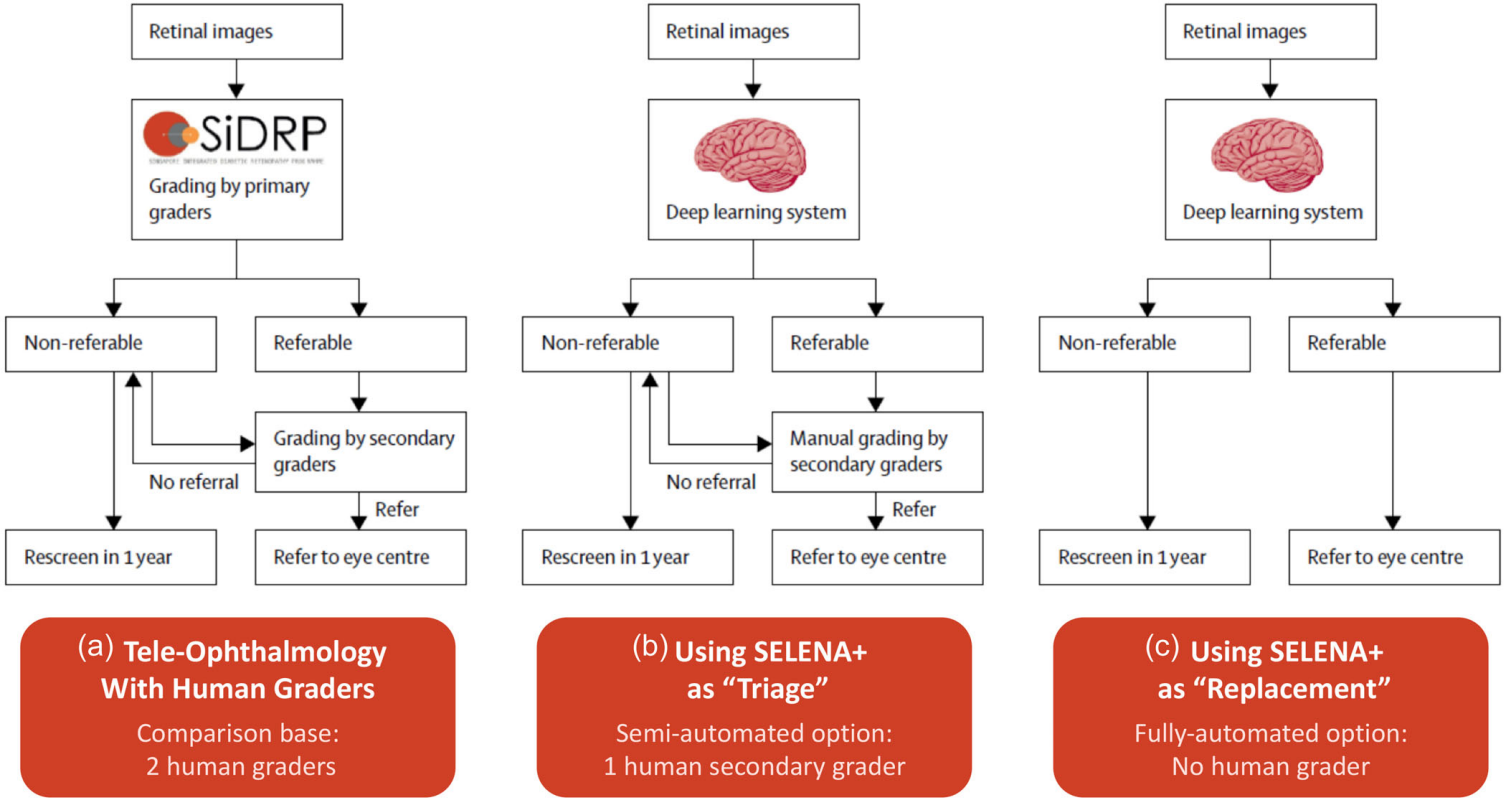
The prior human assessment workflow in the Singapore Integrated Diabetic Retinopathy Program (SiDRP) (a) versus the semi‐automated (b) and fully automated (c) screening models using the SELENA+ deep learning system. Based on economic analysis, the semi‐automated option was the one chosen for full scale deployment. *Source*: Xie et al. [12]

Based on the results of the first and second phase of the field trials, the decision was made to use SELENA+ within the SiDRP workflow in the following way: Automate 100% of the first level assessment that had previously been done by the human technician Level I primary graders; however, retain the human technician Level II secondary (and more skilled) grader to review all cases flagged by the AI system as having one of the three eye diseases being screened for. In essence, this was a semi‐automated approach. Part A of Figure 2 (left side) shows the prior manual workflow for SiDRP inside the central Ocular Reading Center. Part B (middle column) and Part C (right column) show the SELENA+ semi‐automated and fully automated workflow options that were tested and evaluated. The reason for choosing the semi‐automated option that retained the secondary human grader and not the fully automated option is explained in the results discussion below.

### 2.3 Results

There are two aspects to consider while analyzing the results of the efforts described above. The first is the impacts of just implementing SiDRP vis‐a‐viz the eye screening situation prior to this program, and the second is the impact of incorporating the SELENA+ AI system into the ongoing SiDRP program.

Prior to SiDRP, to the extent that people would even go to their own family physician or ophthalmologist for annual diabetic retinopathy screening, turnaround times of 2−3 weeks for delivering the completed screening report were common. Once SiDRP was up and running with the national tele‐ophthalmology infrastructure and centralized image reading and assessment, the reports were almost always generated within a day and often within the hour [13]. Other documented improvements to the diabetic retinopathy screening process included better accuracy in reporting and increased patient satisfaction due to shorter turnaround time to receive a report [24].

A comparative cost analysis of the SiDRP approach to eye screening versus the prior family physician‐based ad‐hoc approach showed a S$144 savings per person screened based on direct medical cost and a somewhat larger S$173 per person savings when considering additional factors comprising total costs. These cost figures are premised on an older adult (55 years old), who continues with screening for the remainder of their lifetime [25]. In summary, the tele‐ophthalmology diabetic retinopathy screening model proved to be cost‐effective and practical because all retinal images were interpreted by trained human technician assessors instead of by medical doctor ophthalmologists [12].

The next step in impact assessment was to estimate how the incorporation of SELENA+ within the SiDRP workflow would further improve performance beyond what had already been achieved with using SiDRP with human technical graders for both the Level I primary and Level II secondary assessments as described above.

**Figure 3:**
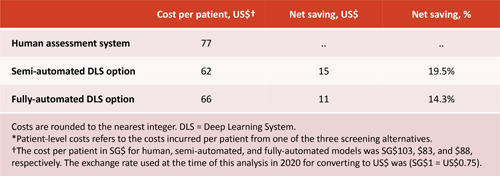
Summary of comparative diabetic retinopathy screening cost per patient with the SELENA+ deep learning system (DLS) considering a semi‐automated and fully automated option versus the base case of human image assessment. *Source*: Xie et al. [12]

A comparative cost analysis published in 2020 estimated potential savings of using SELANA+ within the overall SiDRP workflow considering two options: (i) semi‐automated mode only for Level I primary grading and (ii) a fully automated mode for both Level 1 primary grading and Level II secondary grading [26]. The analysis showed that both the semi‐automated and fully automated use of the SELENA+ AI system were less expensive than the existing baseline of SiDRP with the two level manual grading workflow with human technicians. Surprisingly, their cost analysis and simulation results also showed that the semi‐automated approach would achieve the best economic return for diabetic retinopathy screening. Although the fully automated approach would completely remove human grading and associated technician costs, it also (as of the time of the analysis in 2020) had a higher rate of false positives and would therefore result in more unnecessary referral visits to an ophthalmologist. Hence, the higher costs of retaining Level II graders in the semi‐automated approach is more than offset by the lower ophthalmologist consultation costs given the lower false positive rate achieved using the semi‐automated approach (See Figure 3).

Using SELENA+ in semi‐automated mode, almost 100% of the SELENA+ screening reports are delivered back to the care provider in 30 min or less. In fact, on average, 66% of the reports are delivered within 15 minutes [27].

### 2.4 Future directions for SELENA+

Screening for chronic kidney disease (CKD): The research team that developed SELENA+ and additional research colleagues have demonstrated that the SELANA+ approach—a deep learning algorithm trained on retinal images—can be used in the same neighborhood public polyclinics and community health screening settings to predict the occurrence of CKD with predictive accuracy that has similar performance to that of classic CKD risk factor screening models [28]. This screening approach has the advantage of not requiring the acquisition or processing of blood or urine samples. As such, researchers and clinicians in Singapore's public healthcare system and EyRIS staff are exploring how the retinal image‐based deep learning model can be used to augment already existing approaches for screening of CKD amongst the population of diabetics and other relevant segments of the overall population [29].

Screening for cardiovascular disease (CVD): The current research team is also working together with IHiS to build on SELENA+'s capabilities to trial a predictive risk assessment screening approach for selected CVD based on the analysis of retinal images [30]. Changes in the blood vessels in the retina can provide useful information on an individual's cardiovascular status and risks. For example, narrowed vessels with thickened walls could be due to high blood pressure which is not well controlled. Ongoing exploratory efforts are seeking to determine if analysis of eye retina images can help to identify patients with chronic conditions who are at high risk of getting a heart attack or a stroke above and if so, how this could be used to complement or augment what existing screening methods are able to detect [31].

International expansion: EyRIS has partnered with Topcon Healthcare Solutions, a Japanese company that is a leading provider of eye imaging equipment and related workflow support software, to market and deploy the SELENA+ solution across 18 Asian countries where Topcon already has an existing user base for its eye health products [32]. EyRIS has also obtained regulatory and/or market access approval for using SELENA+ in growing number of countries including Bangladesh, Brazil, European Union, Indonesia, Malaysia, USA, South Africa, and Vietnam and is involved in various stages of pilots, trials, and ongoing usage across these countries [33].

## 3 PREDICTING LONGER TERM INPATIENT READMISSIONS TO GUIDE COMMUNITY CARE INTERVENTIONS FOR PATIENTS WITH COMPLEX CONDITIONS

### 3.1 Objectives

Elderly patients, especially those with multiple care needs, often encounter challenges with taking care of themselves after being discharged from a multiday hospital inpatient stay due to the combined effect of the complexity of their multiple disease conditions and the lack of community‐based care and support services. If such patients end up being readmitted as an inpatient several times over the ensuing 12 months after their “index case” discharge, it places substantial additional load on the limited capacity of the public hospitals as these patients—with a high risk of multiple readmissions, who tend to be 65 years and above—are hospitalized for an average of 10 days each time they are admitted [34].

The first objective of this initiative was to implement a postdischarge community health and social support program focused on admitted inpatients to public hospitals who have the most complex care needs. This includes discharged patients requiring crisis care, living with frailty, who have been high utilizers of public healthcare resources, or in their end‐of‐life phase. It resulted in the “hospital to home” national program started in April 2017 to provide a common nation‐wide community care model targeted at the discharged patients—almost all elderly—with the most complex health and related social support needs [35]. Patients enrolled into the hospital to home (H2H) program receive post‐discharge care and support through home visits and phone follow ups by healthcare professionals that include doctors, nurses, allied health professionals, social workers, and case managers. The objective is to provide adequate community‐based care and support services so that patients with high risk of multiple readmissions can be safely discharged from the hospital in a timely fashion and remain well at home.

The second supporting objective of this initiative was to create a method for identifying which subset of inpatients recently admitted to any public hospital should be placed in this H2H special program because of the combination of their complex post‐discharge support needs and their high risk of returning as an inpatient not just once but several times over the next 12 months.

### 3.2 AI application and total system

The AI application is the prediction model used as a starting point for healthcare staff within each public hospital to identify the subset of inpatients who should be placed in the H2H special program. When this AI model was first deployed in April 2017, concurrently with the start of the H2H program, it was the very first time that an AI prediction model was used across all of the large Singapore public hospitals as part of their daily work related to patient assessments [36].

Input data for the multiple readmissions prediction model includes over one thousand  indicators segmented into three primary categories: sociodemographic characteristics, past hospital utilization information, and information on past medical conditions. Examples of input data indicators include patient age and demographics, number of nonelective inpatient admissions, and total length of hospital stays in the past 2 years, and total number of specialist outpatient visits and emergency department visits in the past 1 year. The output of the prediction model is an indication of those inpatients admitted within the past 24 hours to a particular public hospital who are assessed as having a high risk of multiple readmissions over the next 12 months.

The machine learning method used for the model is a gradient boosting algorithm. While several other ML algorithms were evaluated (logistic regression, random forest, lasso), the gradient boosting algorithm provided both the highest Area Under Curve metric and sensitivity for a prespecified level of positive predictive value (PPV). In addition, model explainability was considered an essential requirement (which is why deep learning systems were not used) and the gradient boosting algorithm has a high degree of interpretability by showing the key indicators via variable importance. This high degree of interpretability has been very helpful in gaining the trust of clinicians during various types of education outreach efforts to familiarize them with the use of the predictive tool.

**Figure 4:**
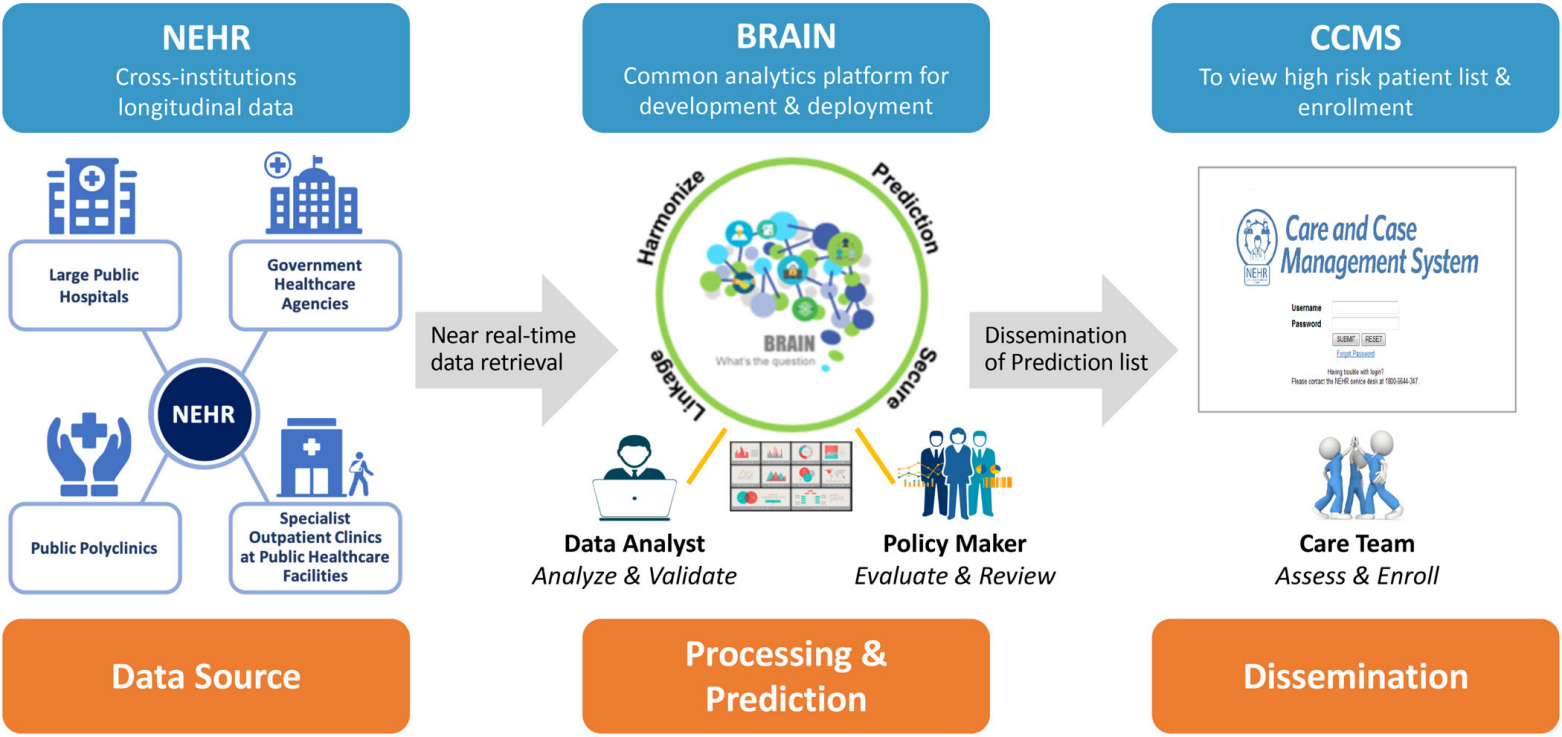
The three major systems required to implement the end‐to‐end workflow for generating and using the multiple readmissions AI prediction model that supports candidate identification for the hospital to home special community support program. *Source*: Ta and Goh et al. [36]

The overall system architecture is shown in Figure 4. All input data for the prediction model originates from the National Electronic Health Record (NEHR) system. All data flows into the Business Research Analytics Insight Network (BRAIN) platform, which is a centralized business intelligence, analytics, and AI processing platform built by IHiS to serve a wide range of data analytics needs across Singapore's public healthcare system. Within BRAIN, a scheduler system triggers the running of the multiple readmissions prediction model on a daily basis for all inpatients recently admitted into any one of the public hospitals and generates patient risk scores.

The BRAIN platform is partitioned into separate secure and near real‐time development and implementation environments with similar system configurations. This substantially reduces the effort and time required to transition from model development, training (and subsequent retraining), and testing to operational deployment and execution.

**Figure 5:**
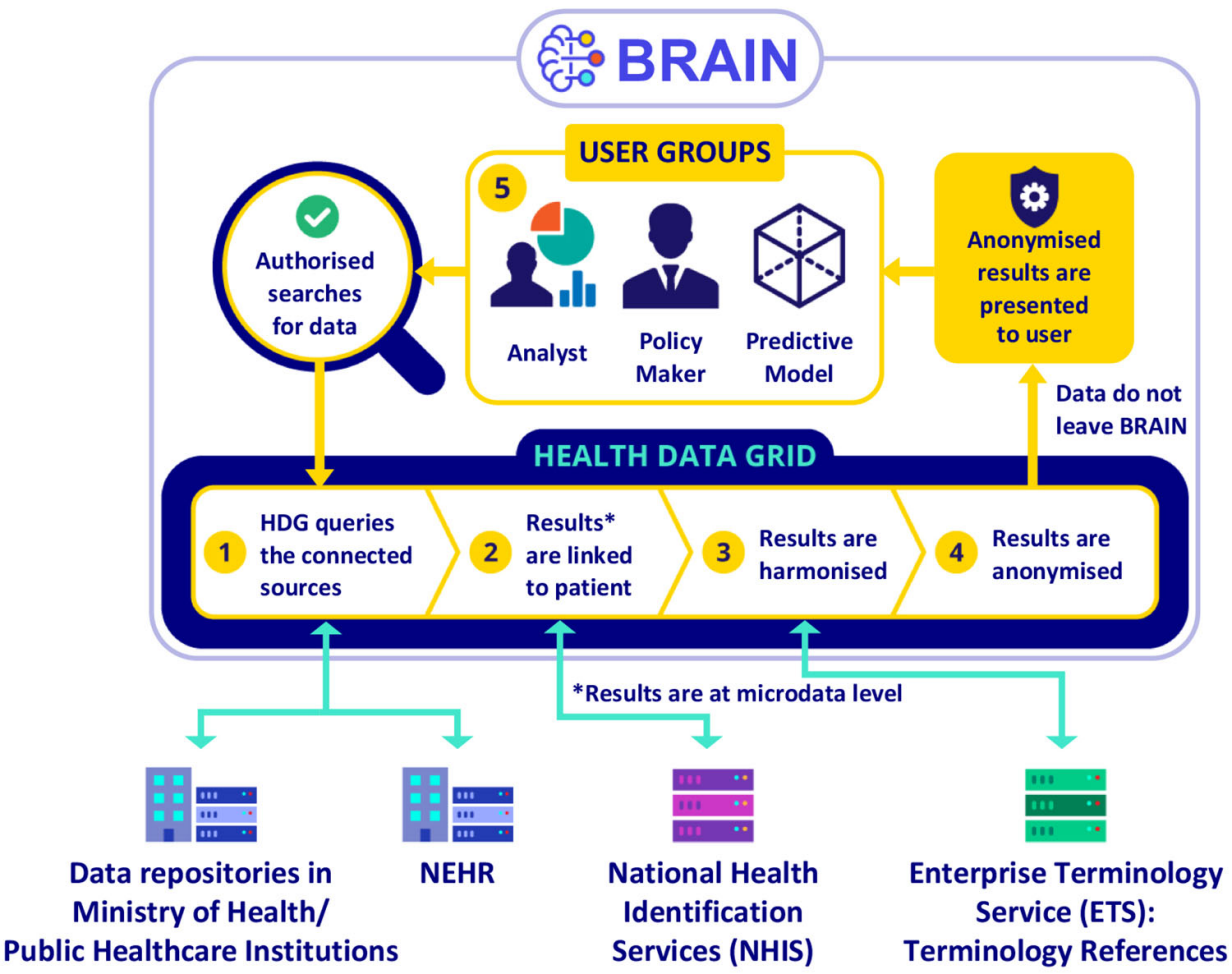
Structure of the Business Research Analytics Insight Network (BRAIN) centralized business intelligence, analytics, and AI processing platform. *Source*: IHiS.

In addition to its link with the National Electronic Healthcare Records system, BRAIN—via the healthcare data grid, the data access and data management part of the platform—is linked to the data warehouses of the three main public hospital clusters and the Ministry of Health (Figure 5). It is also connected to the national system for ensuring accurate linkage across the multiple records of an individual patient and to an Enterprise Terminology Service for harmonizing clinical coding terms used by ICD and SNOMED CT and other sources. This enables BRAIN to support the development and execution of a wide range of AI models (such as most of the examples listed in Table 1), and also to generate and disseminate many types of analytics reports and dashboards.

As shown on the right side of Figure 4, the output of the multiple readmissions prediction model is incorporated into the hospital's Care and Case Management System (CCMS) which provides a standardized view of each patient currently admitted into that hospital and includes their admissions details, demographics, and past medical intervention history within the public health system. The prediction model identifies those with a high risk of multiple readmissions and who also meet other criteria for inclusion in the H2H program.

Using this indication from the prediction model, the nursing team responsible for identifying who should be considered for the H2H program does not have to spend hours combing through all recently admitted patients listed in the CCMS. This is important as only between 10% and 15% of recently admitted inpatients end up being identified by the model as H2H program candidates. Nurses screening for these types of patients can therefore avoid sorting through the other 85%−90% of the recent admits for this particular type of filtering. One important design choice was whether the AI prediction model should include all data that would eventually be available at the end of the current visit (e.g., lab test results, procedures performed, length of stay) or whether the model should be run very shortly after the patient was admitted. Obviously, considering only predictive accuracy for multiple readmissions over the next 12 months, it would be better to run the model at or toward the end of the patient's current stay. However, the H2H clinical team was very clear that they wanted the prediction run as early as possible within the current inpatient stay, within the patient's first 24 hours of being admitted.

Why? For the patients finally selected for the H2H program, the multidisciplinary care team comprising doctors, nurses, therapists, and medical social workers need as much time as possible within the current admissions length of stay to do the following: (i) convince the patient and family members about the benefits of enrolling into the H2H program, (ii) assess the individual patient's post‐discharge needs in order to put together a personalized care plan, and (iii) prepare the patient as well as family members and related family caregivers (e.g., an attendant) for what would need to happen after discharge once the patient is back in their home setting and participating in the program.

Once a patient is discharged from an inpatient stay, the hospital staff know from experience that their ability to communicate with that patient and their family circle, educate them, and influence them about post‐discharge needs and behaviors is dramatically reduced. As those selected for the H2H program would have the most complex care needs and highest risk of multiple readmissions, the imperative was to give the hospital staff more time to engage with the inpatient to get them into the program and be prepared for it.

A second important design choice was where to set the threshold for PPV that guides the AI prediction model in determining which subset of recently admitted inpatients have a sufficiently high probability of multiple readmissions such that they should be recommended as a candidate for the H2H program. If the PPV level was set very high—say at 90% or above—only those patients with the highest probability of having the very highest levels of risk of multiple readmission would be identified—which would result in fewer false positives but also in many more false negatives.

In this context, false negatives turn out to be very expensive over the longer term and place substantial additional load on hospital capacity as these would be people who would not be identified by the model yet who have complex care needs and would very likely end up being readmitted multiple times over the next year. Hence, the H2H clinical care committee advising the design of the model purposely advocated for moderating the PPV level of the predictive model. Working together with the IHiS Data Analytics and AI group, they decided on selecting a PPV of 70 percent to widen the net of candidates to be considered for the H2H program as part of a proactive effort to reduce future inpatient readmissions amongst this set of high‐risk candidates.

**Figure 6:**
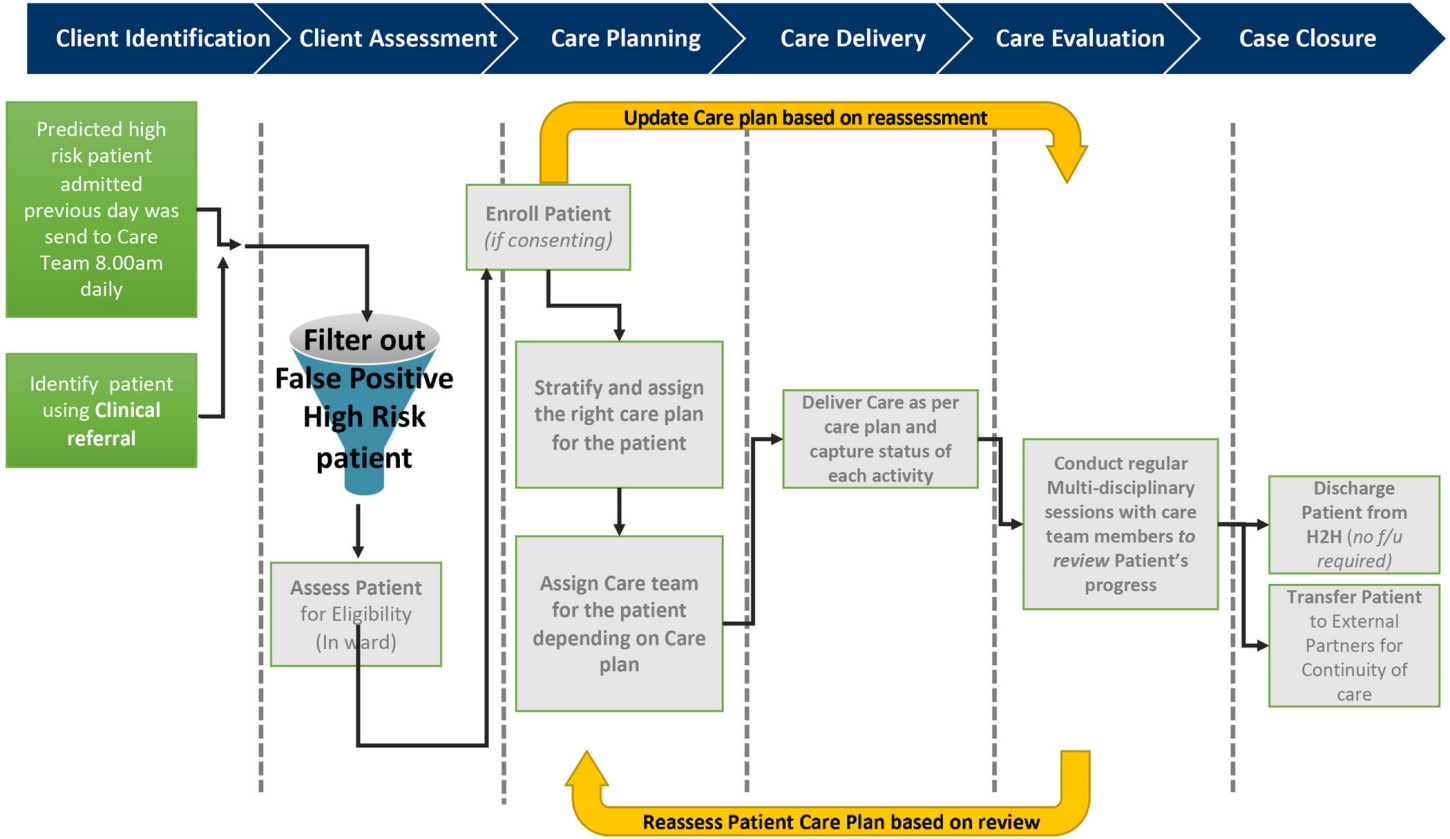
The workflow within each hospital for using the multiple readmission prediction model as part of identifying and evaluating candidates for the Hospital to Home program. *Source*: Ta and Goh et al. [36]

Based on the above strategic decisions, the H2H clinical advisory team and IHiS data scientists worked together to create an operational workflow, which is shown in Figure 6. The AI prediction model output is the primary information used during the patient identification step to identify candidates for the H2H program, though the hospital staff also allow for candidates to be identified from a special clinical referral.

During the patient assessment step, the hospital staff use the predictive model as a sieve to do the first level of filtering. The nurses and other members of the multidisciplinary care team involved in evaluating the candidates for the H2H program do a second round of candidate vetting. As a starting point, they use the AI prediction model's indication of candidates along with all other supporting prior (to the current admission) patient information provided by the CCMS. They also use the most recently available information that has been generated within the current inpatient visit. Using these various sources and their clinical judgment, they filter out what they consider to be the false positives from the prediction model. This leads to the final choice of which of the recently admitted inpatients should be invited into the H2H program.

As part of the care planning step, they engage with those selected patients to seek their consent to participate. If the patient gives consent, the multidisciplinary care team proceeds to create the customized post‐discharge care plan and further engages with the patient and family members to prepare them to execute the H2H care plan.

The care team felt this two‐step approach to initial patient identification followed by a second evaluation and final selection for the H2H program was “near optimal,” clinical practical, and a judicious hybridization of recommendations generated by the AI model and highly contextualized, real‐time human clinical assessment and judgment. This approach balanced consideration of a broader potential candidate pool (because of the moderated value of the PPV to 70%) with an efficient and sustainable approach for filtering (as they only have to vet and evaluate the 10%−15% of recently admitted inpatients selected by the AI prediction model). It also made it possible to do the patient candidate identification, evaluation, and selection very early within the current inpatient stay, and still consider the most recent clinical data from the current stay, though manually and outside of the automated prediction model.

Hospital staff estimate that without using the AI model as a screening tool, nurses tasked with identifying candidates for the H2H program would otherwise have to spend about half of *every day* just manually screening through the entire inpatient list and going round the wards to speak to newly admitted patients and their care team to identify those at higher risk of multiple readmissions. And this screening workload would only increase as the population ages and more elderly people with multiple disease conditions and complex care needs are admitted as inpatients. Without the AI prediction model for multiple readmissions, it would be too time intensive and therefore impractical to identify H2H program candidates on a daily basis within every ward of every public hospital.

### 3.3 Results

This effort demonstrated that an AI predictive model could be practically and successfully incorporated into the daily work of every inpatient hospital ward of every public hospital across all of Singapore's public healthcare clusters [37]. For every one of the patients who consented to participate in the H2H program, the faster screening enabled by the AI prediction model led to that patient having more engagement with the multidisciplinary care team while they were still in the hospital. And as a result of being in the H2H program, they had more home and community engagement and support after discharge.

This effort also demonstrated the viability of using routine data found in the NEHR system to build an AI‐based prediction tool to assist inpatient treatment‐related decision making that could be used by all public hospitals [36]. This in itself was an important result.

This application also demonstrated the feasibility of implementing a patient assessment and follow‐on care delivery workflow in a hospital ward that combines the use of an AI prediction model as a sieve to do the first level of filtering to identify candidates, in combination with clinical staff in the ward doing the second level of evaluation and final decision making by incorporating their clinical judgment and greater awareness of more contextualized and real time considerations. This successful example has paved the way for new initiatives based on this same type of approach.

### 3.4 Future directions for the multiple readmissions prediction model

When the model was initially developed and for the first several years of its usage and refinement, the main sources of data have been the large public hospitals, public polyclinics, specialist outpatient clinics at public healthcare facilities and the relevant healthcare related government agencies (see the left side of Figure 4 under NEHR). As part of the ongoing expansion and enhancement of the model, data from community hospitals, nursing homes, family medicine clinics, and general practitioners potentially can be used to further improve the predictive capabilities of the readmission model as well provide better support for delivering the H2H community‐based care after hospital discharge.

Motivated by the success of the ability to use the NEHR system for creating the multiple readmissions model, IHiS has been working together with various parts of the public healthcare system to use NEHR data to create additional types of AI models that proactively identify health risks in ways that provide decision support to clinicians and patients, especially for chronic disease management.

## 4 MOVING FORWARD AT THE NATIONAL LEVEL AND LESSONS LEARNED

In response to evolving national health strategy and needs, IHiS has been working with all parts of the public healthcare system to create an expanding portfolio of analytics and AI solutions across four broad areas: (i) early stage detection and prevention, (ii) clinical treatment targeting as well as targeting for related special health support programs, (iii) resource optimization within hospitals and polyclinics and across the entire public healthcare supply chain and service delivery network, and iv) administrative support. New systems in the pipeline will also provide added support for health policy formulation for analysts and policy makers within the regional hospital clusters and at the national ministry level.

A sample of this portfolio, including the two applications highlighted in this article and 14 others, is given in Table 1. The two healthcare AI deployments described in this paper (items 1.A and 2.A in Table 1) are amongst the early examples of a steadily growing portfolio of operational AI deployments across the entire public healthcare sector.

As the country moves forward with expanding early stage screening and preventive care for all Singapore residents through a new national initiative called Healthier SG [2], family physicians and GPs will increasingly become the anchor of Singapore's healthcare system. They will need AI risk assessment tools for chronic disease management (e.g., diabetes mellitus/prediabetes, hypertension, lipid disorders, stroke, asthma and chronic obstructive pulmonary disease, and others) [38]. As indicated by the items listed in 1.B of Table 1, progress in this direction is underway and includes an AI‐enabled diagnostic screening effort that will soon be used at scale to identify diabetic patients at risk of kidney failure using the National Diabetes Dashboard. Within the next few years, other AI enabled tools will be field trailed and deployed that will provide health risk scores to GPs and other community‐based medical providers to help them assess the state‐of‐health and disease progression of patients who have selected types of chronic diseases [39]. New tools will also be introduced to expand and improve national screening initiatives for better detection of a broader range of chronic diseases (e.g., heart disease, kidney disease, and others).

Additionally, to enable and enhance sustainable support for an increasing number of AI applications and provide a consistent single source of truth on the patient, IHiS will enhance the BRAIN platform (Figure 5) together with other major healthcare data systems with a common feature store. In addition, IHiS is also standardizing existing and new AI model interfaces with the many other public healthcare IT system via a network of Application Programming Interfaces. This will allow much easier communication and exchange between AI models and the many existing IT systems supporting clinical functions, operations, administration, and community‐based population health efforts. It will also make the BRAIN platform increasingly capable of supporting a wider range of AI applications in the near future.

Since Singapore started its journey from about 2010 onward of implementing the modern generation of data driven machine learning/AI systems, an important lesson that IHiS, and the partner entities comprising the public healthcare sector have learned is that the AI per say cannot take the center stage. Successful production deployments, especially in healthcare settings, are dependent upon intricately weaving AI outputs—predictions, recommendations, resource optimization plans, decision support, and automation control—into daily operational process and their related support systems.

**Figure 7:**
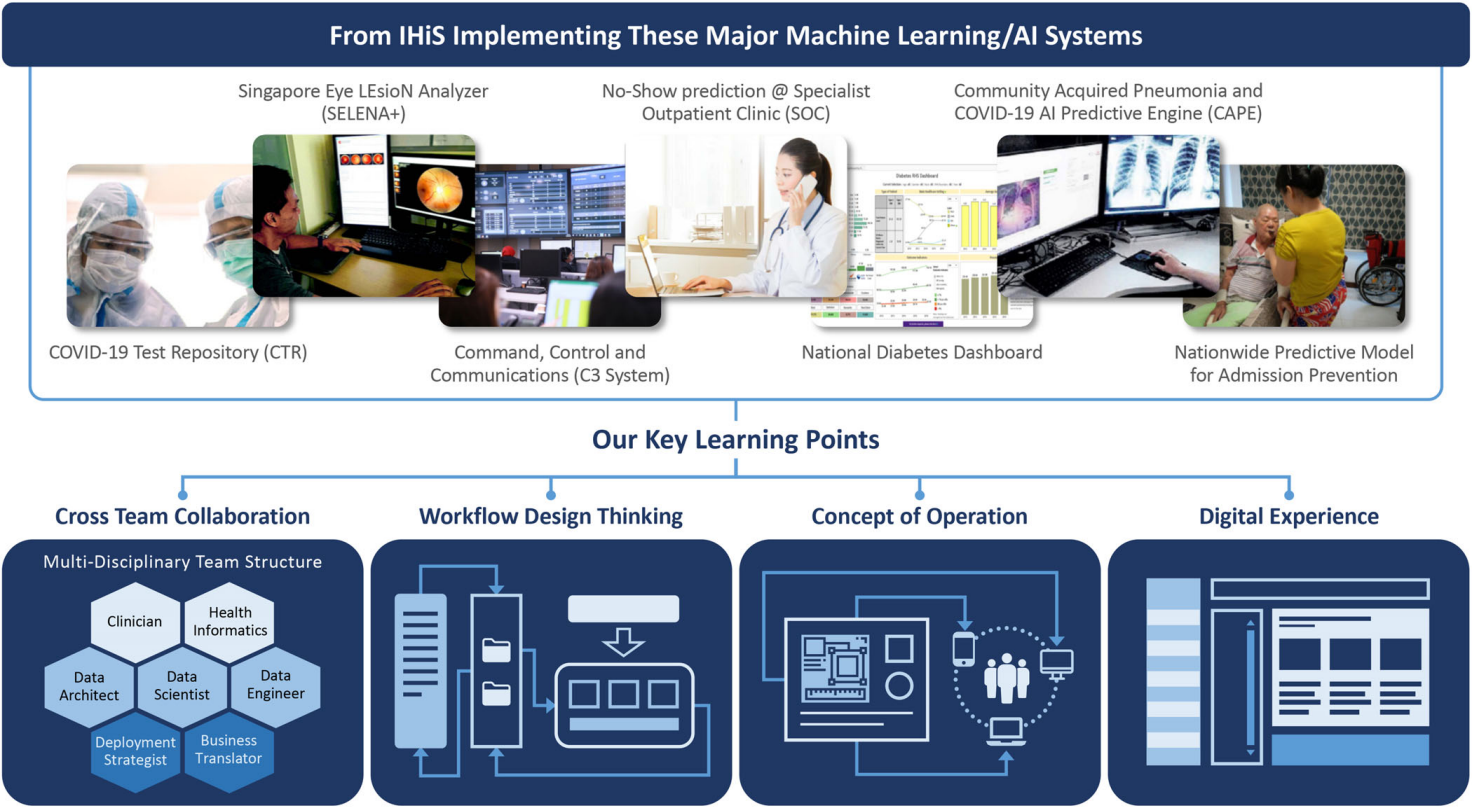
Key lessons learned from Singapore's experience with public healthcare sector AI deployments. *Source*: IHiS.

This has required substantial software engineering efforts that enable the AI model outputs to integrate seamlessly with all other software systems that are part of the relevant workflows. It also requires intense attention on providing a good “digital experience” for all users, including healthcare staff, patients, and their caregivers. As shown in Figure 7, taking the necessary capabilities in AI model development and testing as a given prerequisite, the key to Singapore's public healthcare sector successfully deploying a number of AI and closely related analytics systems to date has been the total embrace of: (i) cross functional collaboration among healthcare service provider partners as well as with other parts of IHiS, (ii) design thinking applied to improved or new workflows, (iii) defining and executing new concepts of operation to implement the workflows, and (iv) improving usability and user experience to provide a better overall approach to digital collaboration and augmentation.

This has also required continuous and collaborative learning with all stakeholders, ongoing efforts to test many different new approaches and methods, and parallel efforts to increase system interoperability and strengthen data security and privacy.

This largely explains why the two AI enabled deployments highlighted in this article took years to plan, create, trial, verify, refine, integrate, and deploy at national scale. This obviously requires a national or regional entity (government or private sector healthcare provider) that can patiently stay the course with such efforts over an extended period of time.

Based on these lessons learned, Singapore will continue its journey to pragmatically and progressively deploy more AI‐enabled systems to support the improvement of healthcare service delivery and to enhance patient engagement in the community as well as in the large public hospital facilities. IHiS, as Singapore's national healthtech agency, will continue to work with all entities that comprise the country's public healthcare service delivery ecosystem to develop and deploy new systems and provide support for the majority of the IT systems and related AI applications.

## AUTHOR CONTRIBUTIONS

**Andy Wee An Ta**: conceptualization (equal); data curation (equal); funding acquisition (lead); methodology (lead); project administration (lead); software (equal); writing—original draft (supporting); writing—review & editing (supporting). **Han Leong Goh**: conceptualization (equal); data curation (equal); funding acquisition (supporting); methodology (lead); project administration (equal); software (equal); writing—original draft (supporting); writing—review & editing (supporting). **Christine Ang**: conceptualization (supporting); data curation (equal); funding acquisition (supporting); methodology (supporting); project administration (equal); software (equal); writing—original draft (supporting); writing—review & editing (supporting). **Lian Yeow Koh**: conceptualization (supporting); data curation (equal); methodology (supporting); project administration (equal); software (equal); writing—original draft (supporting); writing—review & editing (supporting). **Ken Poon**: conceptualization (supporting); data curation (equal); methodology (supporting); project administration (equal); software (equal); writing—original draft (supporting); writing—review & editing (supporting). **Steven M. Miller**: writing—original draft (lead); writing—review & editing (lead).

## CONFLICT OF INTEREST

The authors declare no conflict of interest.

## ETHICS STATEMENT

This article is a practice‐oriented case study description that made extensive use of secondary information sources and also drew upon the professional knowledge of the co‐authors. As such, the creation of this case study article did not involve any formal research study, nor did it involve human participation in a research study. As such, IRB review was not required for this article.

## INFORMED CONSENT

None.

## Data Availability

Data sharing is not applicable to this article as no new data were created or analyzed in this case study description.
